# *In vivo* imaging of cellular proliferation in renal cell carcinoma using 18F-fluorothymidine PET

**Published:** 2014

**Authors:** Peter K. Wong, Sze Ting Lee, Carmel Murone, John Eng, Nathan Lawrentschuk, Salvatore U. Berlangieri, Kunthi Pathmaraj, Graeme J. O’Keefe, John Sachinidis, Amanda J. Byrne, Damien M. Bolton, Ian D. Davis, Andrew M. Scott

**Affiliations:** 1 Department of Surgery and Urology, University of Melbourne, Austin Hospital, Heidelberg, Victoria, Australia; 2 Centre for PET, Austin Hospital, Heidelberg, Victoria, Australia; 3 Ludwig Institute for Cancer Research, Austin Hospital, Heidelberg, Victoria, Australia; 4 Department of Medicine, University of Melbourne, Austin Hospital, Heidelberg, Victoria, Australia

**Keywords:** FDG PET, FLT PET, Renal cell carcinoma

## Abstract

**Objective(s):**

The ability to measure cellular proliferation non-invasively in renal cell carcinoma may allow prediction of tumour aggressiveness and response to therapy. The aim of this study was to evaluate the uptake of 18F-fluorothymidine (FLT) PET in renal cell carcinoma (RCC), and to compare this to 18F-fluorodeoxyglucose (FDG), and to an immunohistochemical measure of cellular proliferation (Ki-67).

**Methods:**

Twenty seven patients (16 male, 11 females; age 42-77) with newly diagnosed renal cell carcinoma suitable for resection were prospectively enrolled. All patients had preoperative FLT and FDG PET scans. Visual identification of tumour using FLT PET compared to normal kidney was facilitated by the use of a pre-operative contrast enhanced CT scan. After surgery tumour was taken for histologic analysis and immunohistochemical staining by Ki-67.

**Results:**

The SUVmax (maximum standardized uptake value) mean±SD for FLT in tumour was 2.59±1.27, compared to normal kidney (2.47±0.34). The mean SUVmax for FDG in tumour was similar to FLT (2.60±1.08). There was a significant correlation between FLT uptake and the immunohistochemical marker Ki-67 (r=0.72, *P*<0.0001) in RCC. Ki-67 proliferative index was mean ± SD of 13.3%±9.2 (range 2.2% - 36.3%).

**Conclusion:**

There is detectable uptake of FLT in primary renal cell carcinoma, which correlates with cellular proliferation as assessed by Ki-67 labelling index. This finding has relevance to the use of FLT PET in molecular imaging studies of renal cell carcinoma biology.

## Introduction

In recent years, positron emission tomography (PET) with ^18^F-fluorothymidine (FLT) has been used to image cellular proliferation. The ability to measure proliferation *in vivo* has several benefits, including the detection of tumour, the non-invasive assessment of tumour grade (1, 2), and evaluation of response to therapy (3-7). A number of tumours, including breast, thoracic, colorectal, soft tissue sarcomas and lymphoma have been studied with FLT PET, with a correlation between FLT uptake and immunohistochemical measurements of proliferation (Ki-67) reported (2, 8-17). However, the role of FLT PET in renal cell carcinoma (RCC) has yet to be established, particularly in terms of correlation with Ki-67 proliferation index (18). Whilst FLT uptake has been shown to increase during sunitinib withdrawal in patients with renal cell carcinoma and other solid malignancies, this was not correlated with post-treatment Ki-67 proliferation index (19).

FLT accumulates in proliferating cells by undergoing phosphorylation by the enzyme thymidine kinase 1 (TK_1_). This leads to intracellular trapping of FLT. The activity of TK_1_ varies during the cell cycle, being highest during the late G1 and S phases of the cell cycle, when preparation for DNA synthesis is occurring. During G0 (quiescent) phase there is very low or no activity of TK_1_. Therefore proliferating tissue, such as tumour, is expected to have uptake of FLT. In addition, malignant cells have been shown to have deregulated TK_1_ activity, leading to increased phosphorylation and accumulation of FLT (20, 21).

A potential advantage of a proliferative tracer compared to the more commonly used PET tracer ^18^F-fluorodeoxyglucose (FDG) is the improved ability to distinguish inflammation from tumour. FDG PET reflects glucose metabolism, which may be increased in both benign inflammatory and malignant tissue. Animal studies comparing FLT with FDG in acutely inflamed or infected tissue have shown that FLT PET is less likely to be falsely positive in these situations (22). *In vitro* studies have demonstrated that FLT PET can measure the antiproliferative effects of tyrosine kinase inhibitors (23). With the introduction of tyrosine-kinase inhibitors for metastatic RCC, such as sunitinib (Sutent®, Pfizer) and sorafenib (Nexavar®, Bayer/Onyx), FLT PET may have the potential to be used to detect a response to these treatments.

The purpose of this study was to determine whether cellular proliferation can be quantitatively imaged in primary RCC by FLT PET. A secondary aim was to compare the uptake of FLT to FDG as measured by maximum standardized uptake values (SUV_max_), and establish whether a correlation exists between FLT uptake, morphologic changes in tumour and cellular proliferation by Ki-67 immunohistochemistry staining in RCC.

## Methods

### Patients

Patients with suspected renal cell carcinoma suitable for nephrectomy were invited to participate in this study. All patients signed informed consent to a protocol which was approved by the Austin Health Human Research Ethics Committee. Patients had FDG PET and FLT PET scans within two weeks prior to surgery. Histopathologic assessment of all tumours was performed for diagnosis and grading, and immunohistochemical staining for the proliferative marker Ki-67.

### PET scan procedure & image interpretation

Synthesis of FDG and FLT occurred on site in our radiochemistry facilities, using an in-house cyclotron (Ion Beam Applications S.A., Louvain-la-Neuve, Belgium) and radiochemistry synthesis techniques as previously described (24, 25). FDG studies were conducted 60 minutes after injection of 370 MBq of FDG. Patients were fasted for at least six hours prior to their scan. FLT scans were conducted on a separate day, with patients not required to fast. 370 MBq of ^18^F-FLT was administered, and scanning occurred after 60 minutes. Standard whole body images (from base of skull to mid thighs) for both PET scans were acquired on the Gemini PET/CT (Phillips Healthcare, Cleveland, Ohio, USA) or Allegro PET (Phillips Healthcare, Cleveland, Ohio, USA) scanner. Attenuation correction was performed from low dose CT scan (Gemini PET scanner), or ^137^Cs point source (Allegro PET scanner).

CT scans and patient history were available to two nuclear medicine specialists. The CT scans were used to identify the region of interest (ROI) for analysis and calculation of standardized uptake values. To improve the accuracy in identifying tumour from excreted radiotracer delayed phase, CT scans were used to exclude the collecting system from the region of interest. The contralateral kidney was used to represent the SUV_max_ of normal kidney. A visual grading system was also used to assess tumours. A score of 0 represented tumour uptake less than normal kidney, 1 representing an isointense tumour, and 2-4 uptake being mild, moderate or markedly greater than normal tissue.

### Histology and immunohistochemistry

The diagnosis of disease was based on histopathology obtained via surgical resection. To determine Ki-67 staining, paraffin blocks tumour were cut into 4 μm sections, dewaxed, and antigen was retrieved in citrate buffer at 100 ºC water bath for 20 minutes. They were then stained with a monoclonal antibody to Ki-67 (rabbit monoclonal IgG antibody, clone SP6, Thermo Fisher Scientific, Fremont, CA, USA) in a 1:50 dilution. Positive and negative controls were obtained, either through the use of highly proliferating reference tissue, or by using an IgG-matched control antibody, or omission of the primary antibody. Areas with the greatest density of staining were chosen for analysis. At 400x magnification, image acquisition software (ImageJ, National Institutes of Health, USA) was used to assist in manually counting at least 500 cells, in three fields within the region of interest. Nuclei with any staining for Ki-67 were considered positive. The Ki-67 proliferative index was defined as the percentage of Ki-67 stained nuclei per total nuclei.

### Statistical analysis

Paired t-tests were used to compare FLT and FDG standardized uptake values. Linear regression analysis was used to compare Ki-67 uptake and SUV. A *P* value of <0.05 was considered to be statistically significant.

## Results

### Patient characteristics

Twenty seven patients (16 males, 11 females; mean age 57.6 years, range 42-77) with suspected primary renal cell carcinoma were recruited into the study. Post operative histology revealed sixteen clear cell RCCs, five papillary RCCs, and one each of sarcomatoid, chromophobe, transitional cell carcinoma (TCC) and oncocytoma. Two patients were found to have benign pathology. Patient characteristics are summarized in Table 1.

**Table 1:** Patient characteristics

Patient	Age	Sex	Size (mm)	Grade	Diagnosis
1	59	M	55	4	Transitional cell
2	63	M	60	3	Clear cell
3	68	F	80	3	Clear cell
4	62	F	100	3	Clear cell
5	63	F	90	3	Clear cell
6	58	M	35	3	Papillary
7	49	M	33	1	Clear cell
8	64	M	33	1	Papillary
9	58	F	18	2	Clear cell
10	46	F	27	2	Clear cell
11	43	F	65	2	Clear cell
12	63	M	30	4	Clear cell
13	77	M	75	3	Clear cell
14	60	M	75	4	Sarcomatoid
15	43	M	75	4	Clear cell
16	62	F	17	3	Papillary
17	47	F	19	2	Papillary
18	42	M	35	2	Clear cell
19	50	M	35	2	Clear cell
20	65	F	20	n/a	Organising haematoma
21	72	M	52	3	Clear cell
22	45	M	20	n/a	benign hamartoma
23	55	M	35	2	Clear cell
24	58	M	32	3	Papillary
25	73	M	35	2	Chromophobe
26	44	F	25	2	Clear cell
27	66	F	35	-	Oncocytoma

### FLT PET scan

**Figure 1:**
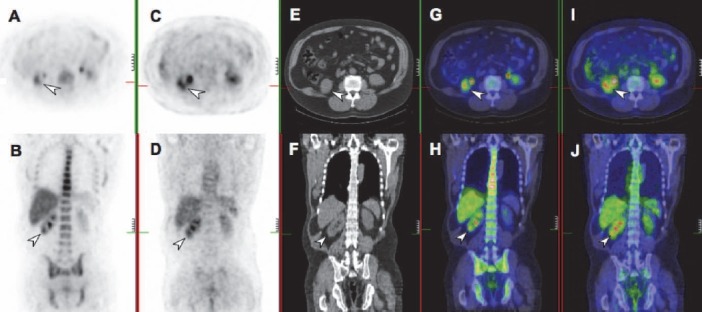
Transaxial and coronal views of a high grade clear cell RCC (arrows). Local recurrence in the right renal bed is seen on FLT PET (A, B), with focalised high uptake. The tumour is also seen in corresponding FDG PET (C, D) and CT (E, F) images. Fused PET-CT images further demonstrate the region of uptake for FLT (G, H) and FDG (I, J)

**Figure 2:**
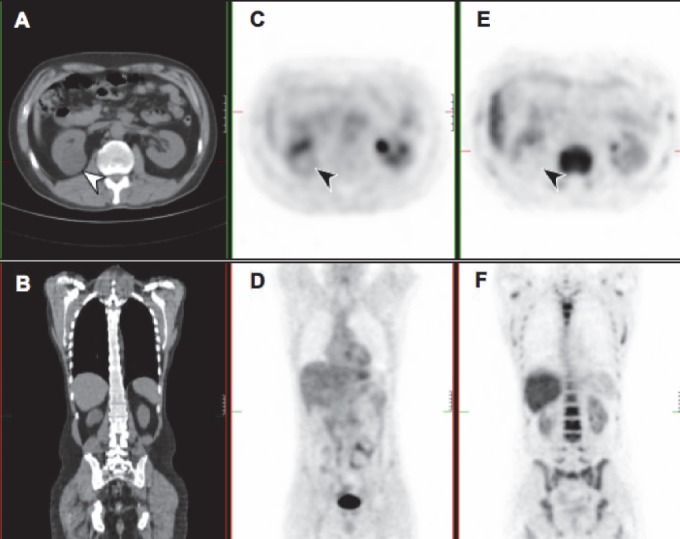
Transaxial and coronal views of a low grade clear cell RCC (arrows). A 35mm mass is seen on the CT image (A, B), with corresponding FDG PET (C, D) and FLT PET (E, F). Uptake in the tumour was less than in surrounding normal kidney

FLT showed uptake in the majority of RCC tumours with a mean tumour SUV_max_ of 2.59±1.27 (range 0.9 – 7.6) (Table 2). The mean tumour SUV_max_ was higher than the mean SUV_max_ of normal kidney, which was 2.47±0.34 (range 1.7 - 3.5), but this did not reach statistical significance. Five tumours had visually discernable uptake greater than the contralateral kidney (visual grading scale 2 – 4) (Figure 1). Nine tumours had a SUV_max_ greater than normal kidney. One of these was a transitional cell carcinoma, having the highest SUV_max_ of 7.6, and highest tumour to normal tissue ratio at 3.6. There was a trend for low grade tumours (Grade 1 – 2; Figure 2) to have a lower mean SUV_max_ at 2.06±0.79, compared to high grade tumours (Grade 3 - 4), which had a mean SUV_max_ of 2.94±1.59). However, this did not reach statistical significance (*P*=0.09). Size and histological RCC subtype did not correlate with FLT uptake.

**Table 2:** PET scan analysis and Ki-67 proliferative index

Patient	Grade	Diagnosis	Ki-67	^18^F-FLT SUV_max_	^18^F-FLT (T/N)	^18^F-FDG SUV_max_	^18^F-FDG (T/N)
1	4	Transitional cell	33.6	7.6	3.6	4.3	1.7
2	3	Clear cell	12.7	2.3	1.1	2.3	1.1
3	3	Clear cell	17.6	2.9	1.0	3.2	1.1
4	3	Clear cell	7.6	1.4	0.6	2.5	1.2
5	3	Clear cell	13.9	2.4	0.9	2.5	1.0
6	3	Papillary	12.1	2.1	0.8	2.4	0.9
7	1	Clear cell	10.6	2.5	1.0	2.5	0.9
8	1	Papillary	12.0	1.9	0.8	3.4	1.2
9	2	Clear cell	7.5	2.5	1.1	2.2	0.9
10	2	Clear cell	15.2	2.6	1.2	2.2	0.8
11	2	Clear cell	4.7	0.9	0.3	1.0	0.6
12	4	Clear cell	28.5	3.9	1.6	3.1	1.5
13	3	Clear cell	18.6	2.2	0.8	2.4	1.0
14	4	Sarcomatoid	21.5	2.6	0.9	6.0	2.4
15	4	Clear cell	36.3	3.6	1.6	4.0	1.7
16	3	Papillary	9.9	3.7	1.1	2.6	1.0
17	2	Papillary	21.8	3.9	1.6	4.6	2.3
18	2	Clear cell	4.2	1.8	0.7	1.8	0.7
19	2	Clear cell	6.6	1.4	0.6	1.5	1.9
20	-	Organising haematoma	n/a	2.3	1.0	2.0	1.3
21	3	Clear cell	13.5	3.4	1.3	2.4	1.4
22	-	Benign hamartoma	n/a	2.2	0.9	2.3	1.0
23	2	Clear cell	6.7	1.9	0.8	1.6	0.9
24	3	Papillary	5.9	1.7	0.6	1.7	1.1
25	2	Chromophobe	2.2	1.6	0.9	1.8	1.2
26	2	Clear cell	5.9	1.7	0.7	2.1	1.2
27	-	Oncocytoma	3.5	2.8	1.3	1.9	1.0

(T/N) – Ratio of SUV_max_ tumour to SUV_max_ normal kidney

### FDG PET scan

FDG PET showed low grade uptake in RCC tumours (Table 2). The mean tumour SUV_max_ was 2.60±1.08 (range 1.0 - 6.0), and was again found to be slightly greater than that of normal kidney, which had a mean SUV_max_ of 2.20±0.54 (range 0.8 - 2.9). There was a greater uptake ratio for tumour to normal tissue in FDG compared to FLT, being 1.21 for FDG, and 1.04 for FLT, although this was not statistically significant (*P*=0.25). A significant difference between the mean SUV_max_ for low grade tumours at 2.25±0.99, compared to high grade tumours at 3.03±1.14 (*P*=0.03) was observed. There was significant correlation between the FLT SUV_max_ and FDG SUV_max_ (*r*=0.52, *P*=0.005).

### Ki-67 proliferative index

**Figure 3:**
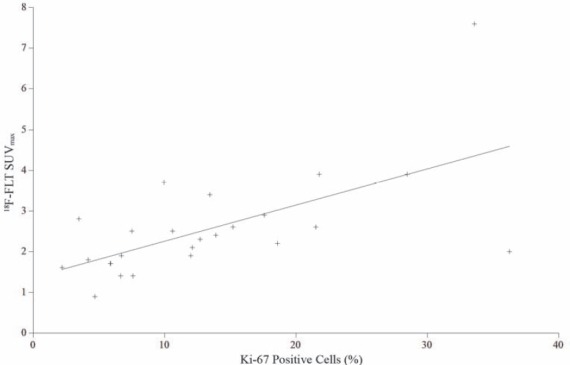
Linear regression analysis of SUV_max_ of ^18^F-FLT and Ki-67 proliferation index. A significant correlation was found (*r*=0.72, *P*<0.0001)

**Figure 4:**
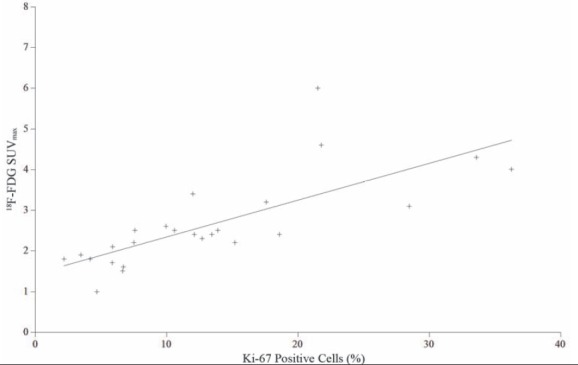
Linear regression analysis of SUV_max_ of ^18^F-FDG and Ki-67 proliferation index. A significant correlation was found (*r*=0.73, *P*<0.0001)

Twenty five tumours were available for analysis using the proliferative marker Ki-67, as no tumour was present in two cases (mean 13.3%±9.1, Table 2). Proliferative index results ranged from 2.1% to 36.2% of cells staining positive. There was a significant correlation between FLT SUV_max_ and Ki-67 (*r*=0.72, *P*<0.0001; Figure 3) and for FDG SUV_max_ with Ki-67 (*r*=0.73, *P*<0.0001; Figure 4).

Ki-67 proliferative index correlated with tumour grade. Low to moderate grade tumours had 8.9 % of cells positive for Ki-67, and high grade tumours 17.8% (*P*=0.009). Tumour stage, histological subtype and size failed to show a correlation with Ki-67 labelling index.

### Extra-renal disease

Although not the focus of this study, one patient was found to have several pulmonary metastases on PET and CT. Six lesions greater than 15 mm were seen on CT, with FDG PET identifying all. FLT PET identified four of these lesions. Semi-quantitative analysis found greater uptake in FDG PET, with a tumour to normal tissue ratio of 6.5 compared to 4.0 in FLT PET. In comparison, the uptake ratio in the corresponding primary tumour was 1.7 for FDG PET and 0.9 for FLT PET.

## Discussion

There is increasing literature on the role of FLT PET in various tumour types looking at tumour detection and non-invasively assessin tumour proliferation and tumour grade. To our knowledge, this is the first study to show detectable uptake of FLT in primary RCC and to correlate uptake with FDG and Ki-67 proliferative index. FLT uptake in primary RCC was observed, and correlated with tumour proliferative index. There was also a trend for high grade tumours to have increased FLT uptake compared to lower grade tumours. Other studies have found a similar correlation between FLT uptake and Ki-67 proliferative index, including glioma, breast cancer, colorectal cancer, lymphoma, and thoracic cancers (2, 9, 11-17, 26). However, there are conflicting reports where a correlation has not been observed (27-29). In a study of 12 patients with breast cancer, 13 of 14 primary lesions were visualised by FLT PET but no correlation between SUV and Ki-67 was found (28). In contrast, another study found a correlation coefficient of 0.79 in 12 breast cancers (14). Similarly, in thoracic tumours, there are at least two reports (27, 29) which failed to observe a correlation, conflicting with other studies (9, 15, 17). Possible explanations have included tumour pathologic diversity (27), and the varying protocols for FLT PET scanning (14).

Due to low physiological uptake in the mediastinum and brain, most FLT PET studies have been focussed on these regions. FLT PET has been shown to have excellent sensitivity for tumour in these regions, even where FLT uptake is less than FDG (10, 27). Although we found similar uptake of FLT and FDG in tumour, most studies have found FLT uptake to be approximately half that of FDG (11, 13, 17, 27, 29). Despite this, the low background uptake of FLT in these areas still allows visualisation of tumour. In our study, integration of anatomic information from CT scans assisted in differentiating FLT uptake in tumour from urinary background uptake.

The nuclear protein Ki-67 can be detected in cells during late G1, S, M, and G2 phases of the cell cycle, but not in the G0 phase. The Ki-67 labelling index has been shown to be an independent risk factor in the prognosis of clear cell RCC (30-32), as well as differentiating low from high grade tumours (32). Our study found low proliferation rates in renal cell carcinoma, as measured by Ki-67 staining. In contrast, Ki-67 levels as high as 85-90% has been observed for other solid tumours (13, 15, 16, 28, 29). Our Ki-67 results are in keeping with prior published studies, and suggest an underlying low proliferative rate in primary RCC. Another possible explanation is that the DNA salvage pathway is not highly active in RCC, which could partly account for the low FLT SUV_max_ found in our study.

Ki-67 uptake was able to differentiate between low and moderate grade tumours and high grade tumours in our study. Visapaa also found that there was increased Ki-67 staining with higher grade of RCC (32). Despite a positive correlation between Ki-67 and FLT uptake, we did not find a significant correlation between FLT uptake and tumour grade, which is most likely due to our sample size. Differentiation between FLT uptake and tumour grade has been reported for other tumours, including soft tissue sarcomas and glioma (12). There is also data that suggest that Ki-67 index may be used as a prognostic factor in RCC. De Risese was the first to report Ki-67 as an independent factor in disease free survival (30). Rioux found that patients with an index greater than 20% had a mean survival of 67 months compared to 42 months (31). In patients with matched tumour stage and grade, Ki-67 may be an additional factor in predicting prognosis.

FDG has been reported to have a sensitivity between 31 and 94% in the assessment of primary RCC (33-36). We found a strong correlation between FLT and Ki-67 (*r*=0.72), which was a similar finding for FDG SUV_max_ as well. The relationship between FDG uptake and proliferation in RCC as measured by FLT PET and Ki-67 proliferative index in renal cell carcinoma has not been previously reported, and our results indicate that the low glucose metabolic rate of primary RCC is matched by a low level of proliferation. Interestingly, one patient in our study had metastatic lesions identified, which all showed prominent uptake of FDG and FLT, highlighting the underlying biologic differences which may exist between primary and metastatic RCC. In many other tumour types, there has also been correlation between FLT uptake, tumour grade and even prognosis, as described in a recent review (37). However, this information has not been evaluated in renal cell carcinoma to date, apart from a pharmacodynamic study which used FLT PET to assess proliferative changes after sunitinib treatment which showed an increase in cellular proliferation as measured by FLT PET, but not with immunohistochemical analysis of Ki-67 index with pathology specimens (19).

## Conclusion

FLT uptake in primary RCC is detectable and correlates with cellular proliferation on Ki-67 biopsy analysis. FLT uptake also correlates with FDG uptake in primary RCC. Further studies are required to define the role of FLT PET in assessing RCC response to treatment and in prognostic assessment, particularly in metastatic disease.
